# Wishes of Children With ADHD

**DOI:** 10.3389/fpsyt.2022.885496

**Published:** 2022-05-02

**Authors:** Emi Furukawa, Ryoko Uchida, Miho Otomo Tatsuki, Margaret Fitts, Gail Tripp

**Affiliations:** Human Developmental Neurobiology Unit and Children’s Research Center, Okinawa Institute of Science and Technology Graduate University, Okinawa, Japan

**Keywords:** wishes, motivation, ADHD, children, Maslow’s hierarchy

## Abstract

Understanding the desires and motivations of children with ADHD is important in helping them thrive. Their inner worlds, however, have not been well captured. The Three Wishes task provides minimal cues and structure to elicit their desires and hopes in an unbiased manner. The wishes of 299 school-aged children with ADHD (193 boys, aged 6–12) were elicited during a research diagnostic assessment. We developed a coding scheme to characterize different aspects of their wishes, including beneficiary, valence, and immediacy. Maslow’s hierarchy of needs, adapted to take account of the participants’ ages, was used to identify the motivations underlying the children’s wishes. As expected, many of the wishes reported were for immediate fulfillment, with many reflecting material desires. Affiliative wishes, highlighting the children’s desire for positive interpersonal relationships, were also common. There was some evidence for self-actualization/self-betterment goals and a small number of altruistic wishes. A word cloud presents the content of the children’s wishes grouped according to this hierarchy. This study highlights the diversity and typicality of the self-reported needs, desires and hopes of children with ADHD. It also serves as a timely reminder of the value of seeking such information directly from children themselves.

## Introduction

Attention deficit hyperactivity disorder (ADHD) is a common neurodevelopmental disorder characterized by elevated levels of inattention, overactivity and impulsivity that impair the affected individuals’ social, academic, and occupational functioning (1). It has been the subject of intense research interest in efforts to better understand and manage the disorder (2). Much of this research has focused on the challenges and difficulties experienced by those with ADHD. Very little attention has been given to the hopes, dreams, and aspirations of those with ADHD.

This impairment-based research emphasis, although driven by the forward goal of improving the lives of those with ADHD, may lead to negative self-perception/lowered self-esteem and reduced expectations of what can be achieved, in the minds of individuals with ADHD, and those with whom they interact. The problems posed by such deficit models are being recognized amid calls to focus on the resources an individual with ADHD is able to recruit (3, 4). Hoogman and colleagues (5) suggest increased knowledge about the positive aspects of ADHD “may aid in treatment and coping with ADHD, reduce stigmatization and increase the quality of life of patients” (p. 81).

To date few researchers have heeded these calls. A review of the literature does show a number of studies have assessed creativity in children and adults with ADHD (5), while the business sector has considered the potential advantages of ADHD symptoms for entrepreneurship (6–8). A small number of studies have focused on the characteristics of successful adults with ADHD (9, 10). In studies of children with ADHD, resilience and protective factors have been considered (11). However, largely absent from this body of research are the perspectives of the children themselves (12). Giving voices to children with ADHD can facilitate understanding of their lived experiences and desires, rather than what others in their lives want for them. In the current study, we begin to address these research gaps by examining the wishes of elementary-age children with ADHD.

A common approach to collecting such information from children is the Three Wishes task. This is a minimally structured technique, used by many clinicians, to elicit information on an individuals’ desires, hopes, goals and concerns in an unbiased manner. While the exact phrasing of the question varies across studies, children are asked to express three wishes, without reference to the likelihood or probability of their occurrence. The technique is seen as providing “ecologically, psychologically and developmentally meaningful data” (p. 914) (13).

When used in research, children’s wishes are recorded and later coded into previously determined categories, the number and nature of which varies depending on the study purpose. A number of studies have focused on the wishes and desires of typically developing children; however, this approach has also been used to study the wishes of children and adolescents facing a range of challenges, including physical illness (14, 15), behavioral and psychological difficulties (16, 17), intellectual disability (18), and disadvantage/abuse (19–21). The Three Wishes task has not previously been used in research studies to assess and document the desires and motivation of children with ADHD.

In child and adolescent populations, the Three Wishes task demonstrates developmental effects (15, 16, 21–26). Typically, younger children make more material wishes (16, 21, 23, 27) while the wishes of older children are less materialistic and immediate, and more abstract and inclusive of others (16, 22, 23, 26). However, within elementary school-aged populations (26, 28) and within adolescent populations (29, 30), the focus of children’s wishes is similar. A number of studies have also identified sex differences in the wishes of children and adolescents. Boys’ wishes are more likely to be associated with pleasure (31), money, power, sport, personal achievement, and possessions (16, 19, 22, 24, 28, 29), social roles (21) and welfare (31, 32). The wishes of girls have been described as more likely to be associated with intimacy, pets, and caring about others (19, 22), family welfare (31), social and family relationships (16, 21), fulfillment of others (29), personal attributes and skills (28), and to be more realistic (17). However, not all studies with this age group report sex differences (25, 26).

There is some evidence that the wishes of children and adolescents experiencing adversity mirror their difficulties. Martinson and Liu (15) reported the wishes of children and adolescents with cancer reflected the impact of their illness on their lives. In comparing the wishes of child psychiatric patients and their peers, Winkley (17) observed those seen by a psychiatrist were more likely to describe wishes that reflected “real problems,” relating to specific difficulties in their lives. Reviewing the wishes of school children with and without learning or emotional problems, Guarnaccia and Vane (16) concluded children experiencing difficulties were “more concerned with personal possessions and relief from the pressures of school than ‘non-problem children”’ (p. 130). Amongst persons with an intellectual disability, Dykens et al. (18) reported associations between participant responses and psychiatric status or CBCL maladaptive behavior. In contrast to these results, Nereo and Hinton (14) found school aged boys with Duchenne’s muscular dystrophy did not make more health-related wishes than their peers or siblings.

In the current study, children meeting the diagnostic criteria for ADHD [as defined by the Diagnostic and Statistical Manual of Mental Disorders (DSM)] were asked a standard question about their wishes at the end of a semi-structured interview. Specifically, they were asked “If you had three wishes and could change three things about your life, what would you change?” The children’s responses were recorded by the interviewing clinician. This data was subsequently extracted for coding, including identification of the motivation behind the children’s wishes, using a version of Maslow’s Hierarchy of Needs. According to Maslow’s theory of human motivation, unmet needs generate wants, desires, and goals that motivate individuals to take actions to obtain them. Maslow’s original hierarchy incorporated five levels; from the essential physiological and safety needs, to higher level needs of love and belonging, esteem, and self-actualization after basic needs are met. Medcalf and colleagues (33) previously used this hierarchy to categorize children’s wishes and dreams for the world. We extended Maslow’s hierarchy for the current study to include the additional motivational categories of avoidance, altruism, and materialistic/hedonistic wants. Many of the categories used in other studies examining children’s wishes are comparable with this extended hierarchy. Other theories of motivation were considered, but not selected given their focus on specific aspects of motivation such as achievement (34), behavioral learning (35), arousal (36), and intrinsic vs extrinsic motivation (37).

In analyzing the wishes of children with ADHD, our goal is to provide insight into their inner worlds through identifying their hopes and desires. We also hope that the information gathered will help us better understand what motivates their behavior. Beyond anecdotal testimonies and clinical observations, we know very little about what motivates children with ADHD (38–40). Such knowledge would help in supporting children with ADHD to thrive and reach their goals. The current study is not designed to evaluate the nature of the wishes of children with ADHD against those of their typically developing peers. Nevertheless, the coding scheme was developed to ensure comparability with those used in previous studies, including those examining typically developing children. We also consider the relationship between age, sex, and socioeconomic status (SES), and the underlying motivations of children’s wishes in the context of existing research with the Three Wishes task. Based on previous studies, we expect to find some differences in the wishes of boys and girls with ADHD. Given the age range of the participants, 6–12 years, marked developmental shifts in the children’s wishes are not expected. While ADHD can create difficulties for the affected child, we do not make specific predictions regarding the degree to which this will be reflected in the children’s wishes.

## Materials and Methods

Ethical approval for the study was obtained from the Okinawa Institute of Science and Technology (OIST) Graduate University Human Subjects Research Review Committee. Participating parents, teachers and children were volunteers and provided written consent.

### Participants

The current study includes data from 299 6–12-year-old children, meeting DSM-IV or DSM-5 diagnostic criteria for ADHD (64.5% boys). One hundred and sixty-two children met the criteria for predominantly inattentive presentation, 12 for predominantly hyperactive/impulsive presentation, and 125 for combined presentation ADHD (see Table 1 for additional participant information). Study inclusion criteria were a diagnosis of ADHD, English as a first language, and no past or current: head injury, neurological disorder, or psychosis. Altogether 317 children met study inclusion criteria, however, 18 children did not provide valid wishes (e.g., “I don’t know,” “I want more wishes”).

**TABLE 1:** Participant demographic and diagnostic characteristics.

	*N* = 299		
	*M*	SD	Range

Age (years)	8.72	1.64	6 – 12
FSIQ	100.32	12.48	68 – 135

		*n* (%)	
Boys		193 (64.5)	
Inattentive/Hyper-Impul/Combined		162 (54.2)/12 (4.0)/125 (41.8)	
Stimulant^a^/Non-stimulant Medication		93 (31.1)/8 (2.7)	
ODD/Mood/Anxiety		10 (3.3)/5 (1.7)/23 (7.7)	
ASD/LD/Other		15 (5.0)/20 (6.7)/18 (6.0)	
Annual Household Income^b^			
< 30K/30K-60K/ > 60K ($)		9 (3.0)/104 (34.8)/167 (55.9)	
Race			
White/Black/Asian/Other^c^		175 (58.7)/32 (10.7)/6 (2.0)/86 (28.5)	

*^a^Children prescribed stimulant medication (n = 93) discontinued its use for at least 48 h prior to participation. ^b^19 (6.4%) participants did not provide income information. ^c^Other included those reporting as “mixed” and “Hispanic.”*

### Recruitment and Assessment

Children were recruited through the OIST Children’s Research Center^1^. English-speaking families living in Okinawa received study information from American/international school personnel, health care professionals, and community support organizations, and volunteered directly. The prefecture of Okinawa is home to a moderate sized English-speaking community. The families participated in multi-method multi-informant research diagnostic assessments. Data from the semi-structured Kiddie Schedule for Affective Disorders and Schizophrenia for School-Aged Children [K-SADS-PL DSM-4 or 5, ADHD, oppositional defiant disorder (ODD) and conduct disorder (CD) sections] (41), parent and teacher ratings of ADHD symptoms from the Conners Behavioral Rating Scale (CBRS) (42) or the Swanson, Nolan and Pelham Rating Scale (SNAP-IV) (43) and observations of the child, during testing and interviews, were used to determine if they met the DSM criteria for ADHD, operationalized as: displaying six or more symptoms of inattention and/or hyperactivity/impulsivity in at least one setting, clear evidence of symptoms in a second setting (e.g., school or clinic), and functional impairment from symptoms. Symptoms were not combined across informants. Parent responses to questionnaires inquiring about a range of internalizing and externalizing difficulties [CBRS or the Child Behavior Checklist (CBCL) (44)], developmental, medical, and academic history questionnaires, and clinical interviews were used to screen for other behavioral and emotional problems and neurological and medical conditions. The ADHD presentations and comorbid conditions reported in the Table 1 are based on the final research diagnosis made after the multi-method multi-informant assessment. Cognitive functioning [Full scale IQ (FSIQ)] was assessed with the Wechsler Intelligence Scale for Children (WISC) version IV (45) or V (46). Assessments were carried out by a United States licensed psychologist, or supervised research staff. All diagnostic decisions were reviewed by at least two doctoral-level clinical psychologists. Demographic data (e.g., age, sex, income, and race) was collected *via* parent completed questionnaires.

### Three Wishes Data Collection

At the end of the semi-structured child interview, participants were asked “If you had three wishes and could change three things about your life, what would you change?” This question was intended, in part, to end the child interview positively, following a discussion of any difficulties the child was experiencing in their daily life. The examiner recorded the children’s responses. These data were subsequently extracted for coding. Some children provided less than three wishes while others provided more. All valid responses were retained for coding and data analysis.

### Coding and Visualization of the Children’s Wishes

The content of the children’s wishes was analyzed in two ways: (1) coding the wishes according to a number of distinct predetermined categories, and (2) graphical representation of the content of the children’s wishes (nouns). For coding and analytic purposes each wish was treated independently.

A scheme to code the children’s wishes was developed based on the codes used in previous studies, population specific questions of interest, experimental literature on motivational processes in ADHD, and through pilot coding of the wishes of 30 participants. A deductive approach was used to organize their wishes, rather than an inductive approach such as thematic analysis. The former approach was selected to address *a priori* interests and facilitate comparisons with previous studies. The coding scheme is presented in Table 2 together with exemplar wishes.

**TABLE 2:** Three wishes codes, exemplar wishes, and code description.

Codes	Exemplar Wishes	Code Description
**Beneficiary**		Beneficiary of a wish
Self	My parents let me do anything I want	
Familiar others	To make my sister really happy	
Strangers/Abstract entity	Money for the poor	
**Immediacy**		Desire for immediate or later wish fulfillment
Immediate fulfillment	I wish I were on holiday now	
Future fulfillment	Playing in the NBA	
**Fantasy**		Whether a wish is fantasy or “real world” based
Pure Fantasy	Be a cat	
Elements of Fantasy	I wish my brother was my age	
Not Fantasy	I want to have more playdates	
**Valance**		Whether a wish is phrased positively (increasing, improving, gaining something) or negatively (decreasing, reducing, stopping something)
Positive	I want to be better at remembering things	
Negative	I get into trouble less	
**Actively negative**		Whether a wish is actively negative, i.e., a wish about something negative happening to self, others, or entities.
Negative wish	Blow up a building	
Not negative wish	(No mention of wanting something negative to occur)	
**Related to ADHD**		Whether a wish is related to the child’s ADHD, i.e., mentions ADHD or ADHD symptoms
Related to ADHD	My ADHD gets better	
Not related to ADHD	(No mention of ADHD or ADHD symptoms)	
**Related to other difficulties**		Whether a wish is related to difficulties a child experiences (other than ADHD) together with the nature of the difficulty
Externalizing problems	Stop throwing stuff when I am angry	
Internalizing problems	Forget how sad I feel	
Academic/cognitive problem	Be better at spelling	
Social difficulties	XX stops getting annoyed with me	
Not related to difficulties	(No mention of difficulties)	
**Impact of Covid-19**		Whether a wish is related to Covid-19 concerns/restrictions
Related to Covid	Corona would be over	
Not related to Covid	(No mention of Covid-19 related concerns or restrictions)	
**Related to current living situation**		Whether a wish is related to requirement for regular moves and/or necessity of living in a foreign country
Related to current living	Move back to United States	
Not related to current living	(No mention of moves or current living situation)	
**Motivation type**		Motivation underlying a wish (single category per wish)
Avoidance	No homework	
Material/Hedonistic	I want an Xbox	
Physiological needs	Sleep better	
Safety/Self-protection	Kids stop bullying me	
Affiliation/Belonging	My friends live nearby	
Self-esteem/Self-actualization	Be good at math	
Betterment of familiar others	Make my sister more popular	
Altruism/Societal betterment	No wars	

*As permission was not requested to publish the children s actual wishes, these represent the types of wishes the children made.

Based on their age and the wording of the question, the children were expected to be the beneficiary of their wishes. We coded for the wish beneficiary, to capture whether they also considered the needs of others in their wishes (47). In general, children are expected to favor immediate wish fulfillment. As children with ADHD show a strong preference for immediate reinforcement in experimental studies (48), we coded for the time frame of their wishes.

Consistent with previous studies all wishes were coded for whether or not they incorporated fantasy or elements of fantasy. Wish valance was also coded. The valence of wishes can be informative in understanding how children want to effect changes in their lives. As the children were diagnosed with ADHD, we coded for whether or not they made wishes directly related to their ADHD/ADHD symptoms. A significant proportion of children with ADHD experience comorbid/associated problems (1), in response, we coded for the presence of wishes related to other difficulties.

Separate coding categories for COVID-19 and the children’s current living situation were included. Some of the children participated after the onset of the pandemic and experienced associated restrictions. Wishes related to their current living-situation were included as participants are English-speaking children living in Japan due to their parents’ work and experience regular family moves. A code for “actively negative” wishes was added after pilot coding indicated the rare, but important, occurrence of such wishes.

Maslow’s Hierarchy of Needs was adapted to code for the motivations underlying the children’s wishes. Given the age of the participating children, the self-esteem and self-actualization categories were combined into a single category. We further added the categories of avoidance, altruistic, and materialistic/hedonistic wants to address the children’s stated desires to avoid challenging situations, their wishes for the benefit of others, and age-appropriate wishes to obtain toys, pets, etc., or engage in activities of interest. These types of wishes do not fit within Maslow’s original five level hierarchy.

Two coders were trained to use the coding system. This included review and explanation of the coding categories, practice coding of children’s wishes, and meetings to identify and resolve coding disagreements. Once trained, one coder (Primary) coded all the children’s wishes while the other (Reliability coder) coded the wishes of 20% of the children, selected at random. Reliability between the two coders was checked during coding, i.e., for every 100 children’s data coded by the primary coder, the data from 20 of these children was independently coded by the reliability coder and the data checked by EF for signs of coder drift. Once all wishes were coded, percentage agreement for each category and overall agreement were calculated. The overall percentage agreement between the two coders was 95% (all coding categories combined). Agreement by category ranged from 88% (fantasy and motivation type) to 100% (actively negative), see Supplementary Table 1. All coding discrepancies were reviewed by the research team. As no systematic differences in the application of the codes were identified, the responses of the Primary coder were used in the data analyses. We considered resolving coding differences through consensus, however, as the wishes of only 20% of the children were double coded, this was not considered appropriate. The coders were blind to the children’s age and sex throughout coding.

The coded responses were summarized using descriptive statistics. A series of chi-square analyses were conducted to examine the associations between the coded wishes and developmental level (age band), child’s biological sex, family SES (income levels), and ADHD presentation. Chi-square analyses were only carried out when the children’s responses were adequately distributed across the available coding choices, i.e., at least 40 wishes (5%) in two or more coding options. Motivation type was examined for associations with age, sex, and SES. Based on previous research, more materialistic wishes might be expected in younger children, boys, and possibly those with lower family SES. Age and ADHD presentation were examined in relation to problem related wishes (non-ADHD specific). We considered that older children and those with Combined presentation ADHD might report more wishes related to any difficulties they were experiencing. The wishes of children with Hyperactive/Impulsive presentation were excluded from this analysis due to small n. Developmental level (age band) was also examined for the fantasy codes as previous reports indicate greater involvement of such elements in the wishes of younger children.

**FIGURE 1:**
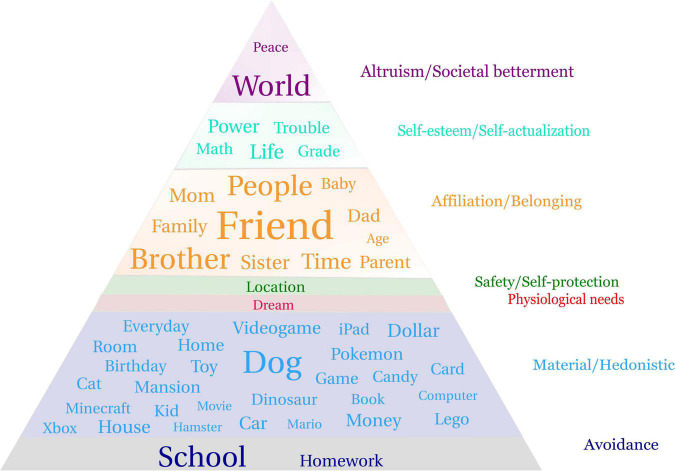
50 most common nouns appearing in the children’s wishes categorized by motivation type using the extended Maslow’s Hierarchy.

Data visualization was undertaken to illustrate the content of children’s wishes in relation to the different motivational categories (i.e., the extended Maslow’s hierachy). This serves to identify the objects and activities populating the children’s wishes, providing a level of specificity not available in the coding system. A word cloud was generated using the 50 most frequently appearing nouns in the children’s wishes, word size indicating the relative frequency of appearance of a given word^2^. Only nouns were included as they described the targets of the children’s wishes with sufficient specificity, whereas common verbs and adjectives tended to be too general (e.g., want, nice). In selecting the nouns, non-specific words were removed (e.g., things). All specific nouns were kept as reported and not combined into broader categories (e.g., Minecraft and Xbox were not subsumed under the noun videogames, dogs and cats not under pets). These nouns were then inputted into the extended Maslow’s Hierarchy of Needs, maintaining the word sizes (Figure 1). In doing this the 50 nouns were reviewed, together with the wishes in which they appeared, to clarify the appropriate category/level of the hierarchy.

**FIGURE 2:**
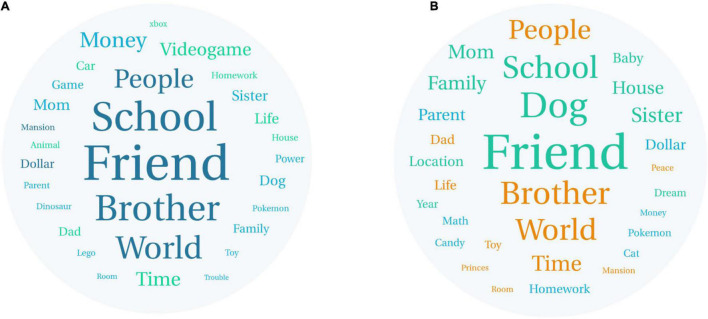
30 most common nouns appearing in the **(A)** boys’ and **(B)** girls’ wishes.

Preliminary analyses generated word clouds separately for the wishes of children in three age bands (age 6–7 *n* = 71; age 8–9 *n* = 136; age 10–12 *n* = 92) representing lower, middle, and upper elementary school. As these three clouds were very similar, data from the entire sample is presented in Figure 1. Word clouds were also generated separately for the 30 most frequent nouns for boys (*n* = 193) and girls (*n* = 106) (Figure 2). Noun frequency tables for the top 50 nouns for the entire sample and the top 30 nouns for boys and girls are presented in the Supplementary Results.

Ahead of generating the word clouds, the data (wishes) were pre-processed using Python in the steps outlined in Supplemental Methods. This included tokenization (breaking sentences into individual words) and removal of punctuation, numerical values, nonsensical text, and stop words (e.g., “a,” “the”). In addition, non-specific words (e.g., thing, stuff, day) were removed from the noun list.

## Results

### Coded Wishes

The nature and frequency of the children’s wishes is summarized in Table 3. A very small percentage of wishes could not be coded for some categories.

**TABLE 3:** Frequency data for the 818 available wishes.

Code	*n*	%
**Beneficiary** Self Familiar others Strangers/Abstract entities Unable to code	754 25 35 4	92.2 3.1 4.3 0.5
**Immediacy** Immediate fulfillment Future fulfillment Unable to code	787 30 1	96.2 3.7 0.1
**Fantasy** Pure fantasy Elements of fantasy Not fantasy	109 103 606	13.3 12.6 74.1
**Valance** Positive Negative Unable to code	663 154 1	81.1 18.8 0.1
**Actively negative** Negative wish Not negative wish Unable to code	3 814 1	0.4 99.5 0.1
**Related to ADHD** Related to ADHD Not related to ADHD Unable to code	8 809 1	1.0 98.9 0.1
**Related to other difficulties** Related to difficulties Not related to difficulties Unable to code Nature of other difficulties (*n* = 77) *Peer relationships* *Sibling relationships* *Relationship with parents* *Relationship with other adults* *Academic/cognitive* *Externalizing behavior* *Internalizing problems* *Other*	77 739 2 11 13 7 1 11 10 5 19	9.4 90.3 0.2 14.9 17.6 9.5 1.4 14.9 13.5 6.8 21.6
**Impact of Covid-19** Related to Covid Not related to Covid	1 817	0.1 99.9
**Related to current living situation** Related to current living situation Not related to current living situation	13 805	1.6 98.4
**Motivation type** Avoidance Material/Hedonistic Physiological Safety/Self-protection Affiliation/Belonging Self-esteem/Self-actualization Betterment of familiar others Altruism/Societal benefit Unable to code	53 402 17 17 163 109 14 33 10	6.5 49.1 2.1 2.1 19.9 13.3 1.7 4.0 1.2

The beneficiary of most wishes were the children themselves (*n* = 754, 92.2%), with a small number of wishes for familiar others (*n* = 25, 3.1%) and abstract entities or strangers (*n* = 35, 4.3%). Likewise, most wishes involved immediate fulfillment (*n* = 787, 96.2%) with only a small number focused on the future (*n* = 30, 3.7%). Approximately a quarter of the children’s wishes involved fantasy, entirely or elements of fantasy based on real world desires, (*n* = 212, 25.9%). The majority of the expressed wishes were positive (i.e., something to be better or more frequent, n = 663, 81.1%) rather than negative (i.e., reduced occurrence or less of something, n = 154, 18.8%). Very few actively negative wishes (i.e., desire for something negative to self or others) were recorded (*n* = 3, 0.4%). The number of wishes specifically related to ADHD or symptoms of ADHD was small (*n* = 8, 1.0%), with only seven children describing at least one wish related to ADHD. More wishes related to other difficulties were identified (*n* = 77, 9.4%). This included 32 wishes related to relationship/interpersonal interaction difficulties, 11 regarding academic/cognitive concerns, 10 about externalizing/behavior problems and five regarding internalizing difficulties. Wishes related to COVID-19 were coded separately; 52 children participated in the study after the beginning of the pandemic. Only one wish was identified that was unequivocally linked to the pandemic (*n* = 1, 0.1%).

Considering the motivation underlying the children’s wishes, almost half of the wishes represented material wants/hedonistic desires (*n* = 402, 49.1%). There were also a substantial number of wishes representing affiliative desires (i.e., wanting to have a better or closer relationship with others, *n* = 163, 19.9%) and self-esteem/self-actualization needs (i.e., wanting to improve oneself or achieve a goal, *n* = 109, 13.3%). A small number of wishes were related to altruistic desires (*n* = 33, 4.4%), betterment of familiar others (i.e., wishing something positive for others, *n* = 14, 1.7%), avoidance (i.e., avoiding objects/places or non-preferred activities, *n* = 53, 6.5%), physiological needs (*n* = 17, 2.1%), and safety/self-protection (*n* = 17, 2.1%).

Chi-square analyses were conducted to examine associations between developmental level (age band) and proportion of wishes related to fantasy, difficulties experienced (“related to other difficulties” code), and motivation type. The chi-square analysis for age band and the frequency of fantasy wishes was significant [χ^2^ (4) = 37.92, *p* < 0.001]. Younger children reported more fantasy wishes than older children. There was also a significant association between age band and motivation type [χ^2^ (14) = 38.75, *p* < 0.001]. Children in the youngest age group reported more materialistic/hedonistic wishes while older children reported more affiliative and altruistic desires. No significant association was observed between age band and the frequency of wishes related to other (non-ADHD specific) difficulties [χ^2^ (2) = 0.83, *p* = 0.660]. Frequency data for the three age bands for the categories of Fantasy and Motivation are presented in Table 4.

**TABLE 4:** Frequency data for the three age groups for the Fantasy and Motivation codes.

		Age 6–7		Age 8–9		Age 10–12	
		*n*	%	*n*	%	*n*	%
Fantasy		*Wish count* = *199*		*Wish count* = *380*		*Wish count* = *239*	
	Pure fantasy	52	26.1	37	9.7	20	8.4
	Elements of fantasy	19	9.5	51	13.4	33	13.8
	Not fantasy	128	64.3	292	76.8	186	77.8
Motivation type		*Wish count* = *198*		*Wish count* = *375*		*Wish count* = *235*	
	Avoidance	6	3.0	36	9.6	11	4.7
	Material/hedonistic	125	63.1	177	47.2	100	42.6
	Physiological	4	2.0	8	2.1	5	2.1
	Safety/self-protection	3	1.5	7	1.9	7	3
	Affiliation/belonging	26	13.1	86	22.9	51	21.7
	Self-esteem/self-actualization	26	13.1	41	10.9	42	17.9
	Betterment of familiar others	4	2.0	6	1.6	4	1.7
	Altruism/societal betterment	4	2.0	14	3.7	15	6.4

Associations between child’s biological sex and family income level were also examined for motivation type. Chi-square analyses showed no significant differences for sex [χ^2^ (7) = 7.38, *p* = 0.390] or income [χ^2^ (14) = 6.64, *p* = 0.948]. Finally, chi-square analysis showed no association between ADHD presentation and the frequency of wishes related to other difficulties [χ^2^ (1) = 2.40, *p* = 0.122].

### Extended Maslow Hierarchy Word Cloud

Figure 1 represents the 50 most common nouns appearing in the children’s wishes, categorized by motivation type (see also Supplementary Table 2). Word size indicates the relative frequency with which the nouns appeared overall and not within each level of the hierarchy. When a noun was used in sentences associated with multiple motivation types, it was placed in the level it was most often associated with. It is important to consider the information presented in Figure 1 together with the information presented in Table 3. Consistent with the motivational coding, words describing friends and family and other affiliation related words (e.g., more “time” with friends and family) feature prominently in the children’s wishes. The nouns appearing in the material/hedonistic level indicate a wide range of desirable objects or interests amongst the children (game, car, toy, dollar, house, Lego etc.), but also pets (dog, cat, hamster), events (birthday), and activity (travel). The targets of the avoidance wishes were school and homework, with school being mentioned many more times than homework. “Dream,” categorized in physiological needs, appeared in children’s wishes wanting a better quality of sleep. “Location”^3^ in the safety level refers to the children’s wish that their living situation would be different (housing needs). The words “power” and “life” were representative of self-actualization wishes, while children also wished to reduce “trouble” and improve their “math” and “grades” which appear as self-esteem related wishes. Some children also wished for a better “world” despite not being asked to make wishes for the betterment of others.

### Boys’ and Girls’ Word Clouds

To visualize the content of the most common wishes of boys and girls with ADHD, separate word clouds of the 30 most common nouns appearing in their wishes were generated (also see Supplementary Table 3). In general, the wishes of boys and girls in the sample appear similar, with friends the most common noun identified for both. Four of the top 5 nouns for boys and girls occupied the same position [friend (1), brother (3), world (4), people (5)]. Second position for boys was occupied by school and for girls by dog. There were some nouns that appeared in the top 30 for boys but not girls, including videogame, time, car, game, and toy. Baby appeared in the top 30 nouns for girls not boys, along with cat and candy. Notably for girls, the nouns homework, family, and parent had a higher relative frequency than for boys, for whom money appeared more frequently.

## Discussion

The current study sought to identify the desires and motivations of children with ADHD through analysis of their wishes. The children were asked to share three wishes about things they would like to change in their lives. Study findings highlight the typicality of the self-reported needs, desires and hopes of elementary age children with ADHD. This is the first study to use the Three Wishes task to better understand the inner worlds of children with ADHD.

Consistent with the instruction to report how they would like to change *their* lives, the beneficiary of the recorded wishes was overwhelmingly the children themselves. For the most part the wishes reported involved immediate fulfillment rather than being future focused. Some children’s wishes, especially those of younger children, involved fantasy. Overall, their wishes appear to be developmentally appropriate (49) and consistent with previous reports of wishes made by typically developing children of similar ages.

As the participating children were all diagnosed with ADHD, their wishes were also coded for whether or not they referenced related difficulties or challenges (15–18). The number of such wishes was low, just over twelve percent summed across all problem categories, including ADHD. The proportion of children making ADHD related wishes was small (2.3%). Amongst “other” problem related wishes (reported by 20% of children), problems with social interactions were the most commonly identified issues. This finding is consistent with research showing elevated rates of social interaction difficulties in children with ADHD (50, 51), and speaks to the desire of children with ADHD to have better interpersonal relationships. Importantly, the wishes of 80% of the children did not include any reference to their difficulties; concerns over their problems do not occupy the everyday thinking of most children with ADHD in this study.

Children’s wishes did not seem to be strongly influenced by their living circumstances. A small number of wishes were related to them living in a foreign country. Only one wish was unequivocally linked to the COVID-19 pandemic. This was a little surprising as a number of children participated in the study after the pandemic began. Given the emerging reports of the negative impact of the pandemic on the mental health of children of this age (52), we did expect to see more wishes in this category. The current result may reflect less life disruption from the pandemic in Japan compared with many parts of the world. It is also possible that the children in the current study are, by and large, coping well in their lives, including with COVID-19 restrictions.

An important focus of the current study was identifying the motivation underlying the children’s wishes. This was achieved by coding the wishes using an extended version of Maslow’s Hierarchy of Needs. This approach has been used previously to analyze the “dreams for the world” of elementary school aged children (33). Changes were made to the original Maslow categories to address the age of the participating children and our interest in their wishes and desires, not just their perceived needs. Almost half of all the children’s recorded wishes were for material benefit/gain or hedonistic/pleasurable activities, which is highly consistent with the children’s ages and previous research. While presented toward the bottom of the hierarchy, this is not meant to imply these are lower ranked or less important wishes. Rather these are simply things the children desire. The next most frequently identified motivation involved affiliation or belonging, with almost 20% of the children’s wishes falling into this category, highlighting the children’s desire for social connections. This is an important finding, children with ADHD tend to experience interpersonal difficulties and receive frequent negative feedback. Our results suggest positive attention and enjoyable time with others, as well as tangible rewards and pleasurable activities, can serve as strong motivators for children with ADHD.

Though fewer in number, several of the children’s wishes reflect their desire to improve their abilities or skills. Some of the children’s wishes also involved the betterment of others and society more generally. This latter result is interesting given the children’s age and that they were asked how they would like to change their *own* lives. Few physiological and safety needs were identified in the children’s wishes, suggesting that the children’s basic needs were being met. The percentage of avoidance wishes was small and reflected the children’s desire to avoid school and homework.

Previous research has identified developmental or age effects, differences between boys and girls, and the impact of psychiatric/learning difficulties on children’s wishes. Consistent with findings with typically developing children, younger children in the current study were more likely than older children to offer wishes that involved fantasy and to make materialistic or hedonistic wishes. Older children, on the other hand, were more likely to make wishes reflecting affiliative and altruistic concerns. In contrast to previous studies, no statistically significant differences between the wishes of boys and girls, or those from different socioeconomic levels, were identified. A review of the most frequently appearing nouns in the wishes of boys and girls identified substantial overlap, but also some notable differences. Videogames and cars were referenced only by boys, while words related to pets and family appeared more frequently among girls.

The Three Wishes task appears to be a useful method for eliciting the hopes and desires of children with ADHD. Participants enjoyed the task and were able to articulate their wishes, with only a small percentage of children being unable to provide any wishes or offering a non-specific desire for more wishes. Given the age range of our sample, this highlights the value of the task for younger children. In the current study we did not specify the time frame for the children’s wishes. In future studies, it would be interesting to ask about children’s wishes for the future, and for others. This would provide more information on children’s career plans and goal setting, and their ability to consider the needs of others. It might also provide a format to elicit information regarding the children’s attitude toward their ADHD. Understanding the perspectives, desires, and goals of children with ADHD, through their words, can help facilitate treatment planning and help them thrive (4, 12, 53, 54).

In interpreting the current data, it is important to acknowledge any study limitations. The wishes of children with ADHD were not directly compared with those of typically developing children, though this was not the study aim. The children in the study were volunteers and as such may be more representative of community samples of children with ADHD than those presenting to specialty clinics, i.e., levels of comorbidity are low and the proportion prescribed medication for symptom management is below that reported in many studies. For several families this was a first assessment for ADHD, likely contributing to lower reported medication use. Thus, the study sample may have influenced the nature of their wishes and should be kept in mind if generalizing the findings to other groups of children with ADHD. That said, all children did meet DSM criteria for ADHD. The reported findings are influenced by the coding scheme utilized. The scheme included elements common to previous studies to facilitate comparisons with this earlier literature. We also included study unique codes (e.g., those related to ADHD and COVID-19). We used word cloud methodology to visualize the content of the children’s wishes, in this case the 50 most frequently appearing nouns. This offers insight into the subject of the children’s wishes but does not represent a thematic analysis. The latter might be considered for future studies using this methodology. In the current study, an *a priori* coding scheme was used to organize their wishes, thus we opted not to identify themes from children’s actual recorded wishes.

The results of the current study offer some important insights as to how we might better support children with ADHD. In clinical practice, it will be important to ask children about their wants, desires, and goals, rather than focusing exclusively on what parents want for their children. Understanding children’s desires may help in motivating them to make behavior changes. This includes attending to positively defined goals, e.g., addressing what children want from their relationships with others, rather than focusing on reducing their social difficulties. We suggest that a goal, rather than impairment, focused approach might serve to motivate and engage children with ADHD in the treatment process. We recommend empirical testing of this proposal.

## Conclusion

The Three Wishes task can be effectively used with children with ADHD to obtain information regarding their hopes and desires. Our findings indicate the wishes of children with ADHD are developmentally appropriate, focusing as they do, on immediacy and material possessions, with some age effects evident in the degree to which they incorporate fantasy and are hedonistic versus affiliative and altruistic. Clearly, the presence of ADHD does not define, or limit, the hopes, and desires of children. This study serves as a timely reminder of the value of including children’s voices in research and clinical practice.

## Data Availability Statement

The raw data supporting the conclusions of this article will be made available by the authors, without undue reservation.

## Ethics Statement

The studies involving human participants were reviewed and approved by OIST Graduate University Human Subjects Research Review Committee. Written informed consent to participate in this study was provided by the participants’ legal guardian/next of kin.

## Author Contributions

EF, RU, and GT conceived and designed the study. MF collected the data. RU and MT performed the coding. EF and RU organized the data, performed the statistical analysis, and prepared the data for visualization. All authors contributed to manuscript preparation and approved the submitted version.

## Conflict of Interest

The authors declare that the research was conducted in the absence of any commercial or financial relationships that could be construed as a potential conflict of interest.

## Publisher’s Note

All claims expressed in this article are solely those of the authors and do not necessarily represent those of their affiliated organizations, or those of the publisher, the editors and the reviewers. Any product that may be evaluated in this article, or claim that may be made by its manufacturer, is not guaranteed or endorsed by the publisher.

## Footnotes

1 OIST is an international university where the official language is English. The OIST Children’s Research Center conducts assessment-based research with English-speaking families (mostly nationals of the United States of America).

2
https://wordart.com/

3 “Location” refers to the specific locality where the children were living. Actual place names were removed to protect the children’s privacy.
