# Supplementary material for: Wishes of Children With ADHD
Source: Front Psychiatry. 2022 May 2;13:885496. doi: 10.3389/fpsyt.2022.885496 (PMC9108194; doi:10.3389/fpsyt.2022.885496)
Supplement: Supplementary file 1 [file Data_Sheet_1.docx]

**Supplemental Methods**

**Supplemental Table 1.** Percentage agreement across two coders for 209 independently coded wishes

| **Code** | **Percentage** | |
| --- | --- | --- |
| Beneficiary | 99% |  |
| Immediacy | 98% |  |
| Fantasy | 88% |  |
| Valance | 99% |  |
| Actively negative | 100% |  |
| Related to ADHD | 100% |  |
| Related to other difficulties | 93% |  |
| Impact of Covid-19 | 99% |  |
| Related to current living situation | 96% |  |
| Motivation type | 88% |  |

**Word Cloud Preprocessing Steps**

The following pre-processing steps were carried out to prepare the children’s recorded wishes ahead of creating word clouds.

1. Text Normalization: removal of capitalization, punctuation, symbols and numerical values.
2. Tokenization: breaking of sentences into individual words (tokens).
3. POS-tagging: labelling of all remaining words according to the Part Of Speech they represent (Noun, Verb, Adjective, Adverb, Pronoun, .etc).
4. Lemmatization: Shortening of words to their meaningful root/base form. (“flying” – “fly”, “families” – “family”, “friends” – “friend”, etc.).
5. Stop word removal: removal of words such as ‘a’, ‘the’, ‘but’, etc. that add little or no meaning to sentences or text.
6. Word Cloud preparation: generation of word clouds using appropriate software. Font size is based on the frequency a word appears in the collated texts, with more frequent words appearing larger.

Programming Language Python’s NLP (NLP Natural language Processing) built-in libraries; SpaCy(<https://spacy.io>), and NLTK(<https://www>.nltk.org) were used for pre-processing text.

WordArt.com. <https://wordart.com/> was used to generate separate word clouds using commonly used nouns.

**Supplemental Results**

**Supplemental Table 2.** Frequency of 50 most common nouns appearing in the 818 children’s wishes used for Figure1.

| **Nouns** | **Frequency** | **Motivation Codes** |
| --- | --- | --- |
| friend | 48 | Affiliation |
| brother | 33 | Affiliation |
| school | 33 | Avoidance |
| world | 30 | Altruism |
| people | 29 | Affiliation |
| dog | 27 | Material |
| time | 25 | Affiliation |
| sister | 24 | Affiliation |
| family | 23 | Affiliation |
| mom | 23 | Affiliation |
| money | 20 | Material |
| dollar | 19 | Material |
| life | 19 | Self-Esteem |
| dad | 15 | Affiliation |
| videogame | 15 | Material |
| house | 14 | Material |
| pokemon | 14 | Material |
| car | 13 | Self-Esteem |
| parent | 13 | Affiliation |
| toy | 13 | Material |
| game | 12 | Material |
| homework | 12 | Avoidance |
| power | 12 | Self-Esteem |
| mansion | 10 | Material |
| room | 9 | Material |
| baby | 8 | Affiliation |
| everyday | 8 | Material |
| kid | 8 | Material |
| lego | 8 | Material |
| trouble | 8 | Self-Esteem |
| candy | 7 | Material |
| dinosaur | 7 | Material |
| home | 7 | Material |
| ipad | 7 | Material |
| birthday | 6 | Material |
| cat | 6 | Material |
| grade | 6 | Self-Esteem |
| math | 6 | Self-Esteem |
| minecraft | 6 | Material |
| location | 6 | Safety/self-protection |
| xbox | 6 | Material |
| book | 5 | Material |
| card | 5 | Material |
| age | 4 | Affiliation |
| computer | 4 | Material |
| dream | 4 | Physiological Needs |
| hamster | 4 | Material |
| movie | 4 | Material |
| mario | 4 | Material |
| peace | 4 | Altruism |

**Supplemental Table 3.** Frequency of 30 most common nouns appearing in the A) boys’ and B) girls’ wishes used for Figure 2.

| **Boys** | | **Girls** | |
| --- | --- | --- | --- |
| **Wishes n = 524** | | **Wishes n = 294** | |
| **Age (years) M = 8.61, SD = 1.64** | | **Age (years) M = 8.92, SD = 1.61** | |
| **Nouns** | **Frequency** | **Nouns** | **frequency** |
| friend | 25 | friend | 23 |
| school | 22 | dog | 15 |
| brother | 21 | brother | 12 |
| world | 19 | world | 11 |
| people | 18 | people | 11 |
| money | 17 | school | 11 |
| time | 15 | time | 10 |
| videogame | 15 | family | 10 |
| dog | 14 | sister | 10 |
| life | 14 | mom | 9 |
| mom | 14 | house | 7 |
| sister | 14 | dollar | 6 |
| dollar | 13 | parent | 6 |
| family | 13 | baby | 6 |
| car | 12 | location | 6 |
| game | 11 | life | 5 |
| dad | 10 | homework | 5 |
| power | 10 | dad | 5 |
| pokemon | 10 | cat | 4 |
| toy | 9 | dream | 4 |
| animal | 8 | year | 4 |
| homework | 7 | math | 4 |
| dinosaur | 7 | candy | 4 |
| house | 7 | toy | 4 |
| lego | 7 | pokemon | 4 |
| mansion | 7 | money | 3 |
| parent | 7 | mansion | 3 |
| room | 6 | peace | 3 |
| trouble | 6 | princes | 3 |
| xbox | 6 | room | 3 |
